# Successful retrieval using ultrathin transnasal esophagogastroduodenoscopy of a significant amount of residual tricyclic antidepressant following serious toxicity: a case report

**DOI:** 10.1186/1865-1380-6-39

**Published:** 2013-10-22

**Authors:** Masato Miyauchi, Makiko Hayashida, Hiroyuki Yokota

**Affiliations:** 1 Department of Emergency and Critical Care Medicine, Nippon Medical School, Tokyo, Japan; 2 Department of Legal Medicine, Nippon Medical School, Tokyo, Japan

**Keywords:** Tricyclic antidepressant, Gastric lavage, Endoscopy, Transnasal esophagogastroduodenoscopy

## Abstract

**Background:**

In Japan, ultrathin transnasal esophagogastroduodenoscopy (EGD) with a 4.9-mm diameter endoscope (Olympus XP260) is routinely used to examine the upper gastrointestinal tract. This procedure does not require sedation and does not affect vital signs. Gastric lavage is not empirically employed in the management of all poisoning patients. It is considered only for potentially life-threatening overdoses when the procedure can be performed within 1 h of ingestion of the poison. However, there are no absolute indications for gastric lavage. EGD may increase the indications, efficiency and safety of gastric lavage in poisoning patients.

**Findings:**

A 35-year-old female was admitted to our emergency department 2 h after ingesting multiple drugs, including a critical dose of the tricyclic antidepressant (TCA) amitriptyline, at which time she was confused and had a Glasgow Coma Scale score of 8 (E1V2M5). Endotracheal intubation was performed. To confirm the type of TCA and in order to determine whether gastric lavage was required, we decided to perform EGD. Endoscopy demonstrated adherence of residual drugs to the stomach wall, in a soluble form and not as a mass. Hence, gastric lavage was performed via the EGD to avoid passage of these drugs into the small bowel. The patient was extubated on day 2, without the development of complications such as aspiration pneumonia, and was discharged on day 5.

**Conclusion:**

EGD may be useful in poisoning patients for determining the amount of residual drug in the stomach, also allowing direct observation of the effectiveness of gastric lavage.

## Findings

### Introduction

Tricyclic antidepressant (TCA) overdosage is a common cause of drug poisoning [1], which can have adverse cardiovascular, respiratory and neurological effects [2]. TCA overdosage may cause fatal cardiovascular toxicity, detected by electrocardiographic abnormalities, arrhythmias and hypotension [3]. Such patients are managed by supportive treatment, including gastric decontamination. However, this procedure has the potential to cause serious side effects, such as hypoxia, laryngospasm, perforation of the gastrointestinal tract, electrolyte abnormalities and aspiration pneumonia [4]. According to one set of guidelines [Guidelines in Emergency Medicine Network (GEMNet]) for TCA poisoning [5], gastric lavage may be considered for potentially life-threatening overdoses only when it can be delivered within 1 h of poison ingestion. However, there is no evidence completely excluding the utility of gastric lavage.

In Japan, ultrathin transnasal esophagogastroduodenoscopy (EGD) with a 4.9-mm diameter endoscope (Olympus XP260) is routinely used to examine the upper gastrointestinal tract. This procedure does not require sedation and can be performed with minimal effects on the patients’ vital signs [6]. EGD can be used to directly observe gastric contents and the lavage procedure. Thus, ultrathin EGD is an effective diagnostic tool that can also be used during gastric lavage treatments without complications.

## Case report

**Figure 1:**
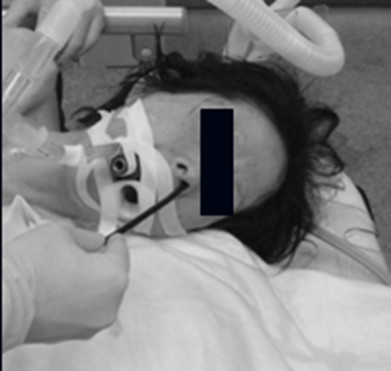
During the procedure, the ultrathin EGD was inserted through the left nostril.

**Figure 2:**
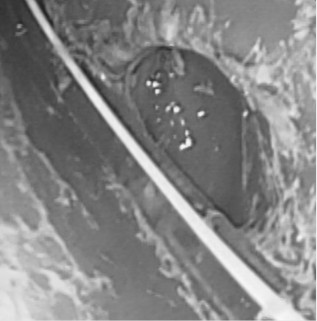
**Endoscopic image showing residual drugs adherent to the stomach wall (*arrows*).** The *arrowhead* indicates the nasogastric tube.

A 35-year-old, 40-kg female with a history of depression was transported to our emergency department by ambulance 2 h after ingestion of amitriptyline (58 × 25-mg tablets; total intake 36 mg/kg), etizolam (36 × 0.5-mg tablets; total intake 0.45 mg/kg) and triazolam (10 × 0.25-mg tablets; total intake 0.06 mg/kg). There was no evidence of vomiting or seizures prior to arrival. Her blood pressure was 110/72 mmHg, heart rate was 83 beats/min, respiratory rate was 19 breaths/min and temperature was 35.3°C. Initial blood gas analysis while breathing 100% oxygen showed a pH of 7.365, arterial carbon dioxide tension (PaCO_2_) of 44.1 mmHg, arterial oxygen tension (PaO_2_) of 317 mmHg, actual bicarbonate level of 24.6 mmol/l (normal range, 22 to 32 mmol/l) and base excess of -0.4 mmol/l (-2 to 2 mmol/l). She was confused and had a Glasgow Coma Scale score of 8 (E1V2M5). Endotracheal intubation was performed and mechanical ventilation was initiated. Electrocardiography showed a QRS interval of 0.104 s. Biosite Triage assays were positive for TCA and benzodiazepine. To confirm the type of TCA and in order to determine whether gastric lavage needed to be performed, we decided to perform EGD. Consent for the endoscopy was provided by the patient’s husband prior to the procedure. Nasal anesthesia was achieved by inserting a catheter coated with 2% lidocaine and 1:5,000 epinephrine gel into the nose for 5 min. She was placed in the left-lateral decubitus position, and the endoscope (lubricated with 2% lidocaine gel) was slowly advanced transnasally (Figure 1). The EGD procedure did not require additional sedation and did not affect her vital signs. Endoscopy demonstrated adherence of residual drugs to the stomach wall in a soluble form. There was, however, no solid material in the stomach (Figure 2). A nasogastric tube was inserted, and gastric lavage was performed by passing water through the tube. The residual drugs were soluble in water and were easily aspirated via the nasogastric tube and the endoscope. The lavage procedure was directly observed via the endoscope to ensure that the gastric contents did not pass into the small bowel. A total of 1,000 ml of water was required to clear the residual drugs from the stomach.

After completion of the gastric lavage, a single dose of activated charcoal was administered though the nasogastric tube. Electrocardiography showed normal QRS and QTc intervals. No additional treatment, such as hypertonic sodium chloride infusion or systemic alkalinization, was administered. The patient was extubated on day 2 without the development of aspiration pneumonia. She was discharged without neurological deficits on day 5.

## Discussion

TCA overdosage can affect the cardiovascular, respiratory, and central and autonomic nervous systems. Many patients require intensive care support or hospital admission [7]. Their management is associated with several difficult issues. The treatments recommended by the GEMNet for TCA overdosage include gastric lavage, activated charcoal administration and bicarbonate infusion. The recommendations also state that gastric lavage should be considered only when it can be performed within 1 h of drug ingestion, in the presence of a protected airway and when the overdosage is potentially life-threatening. Yet, clinical studies have strongly suggested that there is no benefit to performing gastric lavage in this setting. However, it has also been reported that there may be significant drug residue in the stomach at >1 h after drug overdosage [8]. This is because TCA poisoning is associated with gastric hypomotility and a marked delay in gastric emptying due to direct drug effects and stress [9]. Besides, there is no definitive evidence in the position paper update conclusively excluding the beneficial effects of gastric lavage [10]. Identification of residual drug content in the stomach would help to determine the need for gastric lavage. Although computed tomography is used to identify large numbers of tablets in the stomach [11], its use is limited in cases in which a variety of tablets have been ingested. Previously, endoscopy was used for the diagnosis and therapy of a case of gastric bezoars [12].

Although endoscopy in poisoning patients should only be used after airway protection, the EGD procedure does not require additional sedation and does not affect vital signs. In cases of life-threatening overdosage, it is important to determine the amount of residual drug in the stomach. The usefulness and efficacy of gastric lavage in poisoning patients has been debated for a long time [13,14]. In our case, EGD confirmed the gastric contents and facilitated performance of gastric lavage, preventing further passage of drugs to the small bowel by aspiration of gastric contents. Although our patient was intubated and sedated, EGD can also be performed in unsedated patients without any detrimental effects on vital signs.

It is sometimes necessary to determine the amount of residual drug in the stomach in poison patients after arrival at the emergency department. EGD may be a useful procedure to achieve this. Although clinical evidence is lacking regarding the effectiveness of gastric lavage in these patients, in our case using EGD achieved confirmation of the presence of residual drugs and their appropriate clearance from the stomach.

## Conclusion

EGD appears to be a useful and safe procedure for confirmation of gastric contents and the effectiveness of gastric lavage in poison victims.

## Abbreviations

EGD: Ultrathin transnasal esophagogastroduodenoscopy; TCA: Tricyclic antidepressant; GEMNet: Guidelines in Emergency Medicine Network.

## Competing interests

The authors declare that they have no competing interests.

## Authors’ contributions

MM carried out the study design, date interpretation, participated in the sequence alignment and drafted the manuscript. MH carried out date analysis. HY carried out final approval of the article. All authors read and approved the final manuscript.
